# Supplementary material for: Effects of inbreeding and elevated rearing temperatures on strategic sperm investment
Source: Behav Ecol. 2024 Jun 5;35(4):arae044. doi: 10.1093/beheco/arae044 (PMC11187721; doi:10.1093/beheco/arae044)
Supplement: arae044_suppl_Supplementary_Material [file arae044_suppl_supplementary_material.docx]

**Supplementary Material for**

Effects of inbreeding and elevated rearing temperatures on strategic sperm production

Meng-Han Joseph Chung^1^*, Md Mahmud-Al-Hasan^1^, Michael D. Jennions^1,2^, and Megan L. Head^1^

1. Division of Ecology and Evolution, Research School of Biology, Australian National University, Canberra, Australian Capital Territory 2601, Australia

2. Stellenbosch Institute for Advanced Study (STIAS), Wallenberg Centre, Stellenbosch University, Stellenbosch 7600, South Africa

*Meng-Han Joseph Chung ([chungmenghan@gmail.com](mailto:chungmenghan@gmail.com))

***Part 1 – Sample size for each manipulated variable***

| Inbreeding status | Rearing temperature | Social environment | *n* |
| --- | --- | --- | --- |
| Outbred males | Warm  (*n* = 85) | No female | 28 |
|  |  | Unrelated female (outbred) | 28 |
|  |  | Related female (outbred) | 29 |
|  | Control  (*n* = 88) | No female | 29 |
|  |  | Unrelated female (outbred) | 31 |
|  |  | Related female (outbred) | 28 |
| Inbred males | Warm  (*n* = 107) | No female | 28 |
|  |  | Unrelated female (outbred) | 27 |
|  |  | Unrelated female (inbred) | 26 |
|  |  | Related female (inbred) | 26 |
|  | Control  (*n* = 103) | No female | 27 |
|  |  | Unrelated female (outbred) | 24 |
|  |  | Unrelated female (inbred) | 26 |
|  |  | Related female (inbred) | 26 |

***Part 2 – Statistical outputs from models with or without non-significant interactions***

**2-1 Likelihood that we extracted sperm from the male**

1. Initial model including the interaction

|  | | | Estimate | | *SE* | χ²_1_ | | *P* |
| --- | --- | --- | --- | --- | --- | --- | --- | --- |
| Intercept (inbred, control) | | | 9.546 | | 2.515 | 14.401 | | **<0.001** |
| Body length (standardised) | | | -0.036 | | 0.561 | 0.004 | | 0.948 |
| Adult age at testing (standardised) | | | 0.409 | | 0.944 | 0.188 | | 0.665 |
| Male inbreeding status (outbred) | | | 0.415 | | 2.993 | 0.019 | | 0.890 |
| Temperature (warm) | | | -0.147 | | 1.319 | 0.012 | | 0.911 |
| Male inbreeding status (outbred) * Temperature (warm) | | | 21.112 | | 18799.063 | <0.001 | | 0.999 |
| Random effect | Variance | *SD* | | Number of groups | | |  |  |
| Brood ID | 66.270 | 8.141 | | 117 | | |  |  |

1. Final model excluding the non-significant interaction

|  | | | Estimate | | *SE* | χ²_1_ | | *P* |
| --- | --- | --- | --- | --- | --- | --- | --- | --- |
| Intercept (inbred, control) | | | 9.381 | | 2.567 |  | |  |
| Body length (standardised) | | | -0.130 | | 0.558 | 0.055 | | 0.815 |
| Adult age at testing (standardised) | | | 0.561 | | 0.909 | 0.381 | | 0.537 |
| Male inbreeding status (outbred) | | | 1.186 | | 3.026 | 0.154 | | 0.695 |
| Temperature (warm) | | | 0.690 | | 1.185 | 0.339 | | 0.560 |
| Random effect | Variance | SD | | Number of groups | | |  |  |
| Brood ID | 70.02 | 8.368 | | 117 | | |  |  |

**2-2 Changes in sperm production rates in response to different social environments**

1. Initial model including all 2-way interactions

|  | | | Estimate | | *SE* | χ² (df) | | *P* |
| --- | --- | --- | --- | --- | --- | --- | --- | --- |
| Intercept (inbred, control, no female) | | | 358.437 | | 12.435 | 830.810 (1) | | **<0.001** |
| Body length (standardised) | | | 18.899 | | 4.176 | 20.481 (1) | | **<0.001** |
| Adult age at testing (standardised) | | | -24.959 | | 4.355 | 32.840 (1) | | **<0.001** |
| Male inbreeding status (outbred) | | | -10.845 | | 15.862 | 0.468 (1) | | 0.494 |
| Temperature (warm) | | | -22.995 | | 14.985 | 2.355 (1) | | 0.125 |
| Social environment (unrelated female) | | | 29.628 | | 15.997 | 3.449 (2) | | 0.178 |
| Social environment (related female) | | | 11.943 | | 16.328 |  | |  |
| Male inbreeding status (outbred) * Temperature (warm) | | | 21.590 | | 15.154 | 2.030 (1) | | 0.154 |
| Male inbreeding status (outbred) * Social environment (unrelated female) | | | -38.153 | | 18.107 | 7.028 (2) | | **0.030** |
| Male inbreeding status (outbred) * Social environment (related female) | | | 6.625 | | 18.636 |  | |  |
| Temperature (warm) * Social environment (unrelated female) | | | 10.132 | | 17.812 | 1.792 (2) | | 0.408 |
| Temperature (warm) * Social environment (related female) | | | -14.209 | | 18.100 |  | |  |
| Random effect | Variance | *SD* | | Number of groups | | |  |  |
| Brood ID | 805 | 28.37 | | 113 | | |  |  |
| Residual | 4137 | 64.32 | |  | | |  |  |

1. Final model excluding the non-significant interactions

|  | | | Estimate | | *SE* | χ² (df) | | *P* |
| --- | --- | --- | --- | --- | --- | --- | --- | --- |
| Intercept (control, inbred, no female) | | | 354.029 | | 10.815 | 1071.502 (1) | | **<0.001** |
| Body length (standardised) | | | 18.736 | | 4.175 | 20.138 (1) | | **<0.001** |
| Adult age at testing (standardised) | | | -25.563 | | 4.385 | 33.986 (1) | | **<0.001** |
| Temperature (warm) | | | -13.379 | | 7.646 | 3.062 (1) | | 0.080 |
| Male inbreeding status (outbred) | | | -0.674 | | 14.215 | 0.002 (1) | | 0.962 |
| Social environment (unrelated female) | | | 34.363 | | 13.181 | 8.009 (2) | | **0.018** |
| Social environment (related female) | | | 3.709 | | 13.429 |  | |  |
| Male inbreeding status (outbred) * Social environment (unrelated female) | | | -38.072 | | 18.035 | 7.195 (2) | | **0.027** |
| Male inbreeding status (outbred) * Social environment (related female) | | | 7.344 | | 18.610 |  | |  |
| Random effect | Variance | *SD* | | Number of groups | | |  |  |
| Brood ID | 902.8 | 30.05 | | 113 | | |  |  |
| Residual | 4085.6 | 63.92 | |  | | |  |  |

1. Pairwise comparison for the significant interaction between male inbreeding status and social environment

Inbred males:

| Contrast | Estimate | *SE* | df | *t* ratio | *P* |
| --- | --- | --- | --- | --- | --- |
| No female – Unrelated female | -34.360 | 13.2 | 280 | -2.600 | **0.027** |
| No female – Related female | -3.710 | 13.5 | 303 | -0.275 | 0.959 |
| Unrelated female – Related female | 30.650 | 13.7 | 299 | 2.245 | 0.066 |

Outbred males:

| Contrast | Estimate | *SE* | df | *t* ratio | *P* |
| --- | --- | --- | --- | --- | --- |
| No female – Unrelated female | 3.710 | 12.3 | 257 | 0.302 | 0.951 |
| No female – Related female | -11.050 | 12.9 | 294 | -0.854 | 0.670 |
| Unrelated female – Related female | -14.760 | 12.6 | 278 | -1.169 | 0.473 |

Contrast (Inbred males – Outbred males) in three social environments

| Social environments | Estimate | *SE* | df | *t* ratio | *P* |
| --- | --- | --- | --- | --- | --- |
| No female | 0.674 | 14.3 | 247 | 0.047 | 0.962 |
| Related female | -6.669 | 14.4 | 251 | -0.463 | 0.644 |
| Unrelated female | 38.747 | 14.3 | 250 | 2.714 | 0.007 |

**2-3 Changes in sperm production rate of inbred males in response to different female inbreeding status and female relatedness**

1. Initial model including the interaction

|  | | | Estimate | | *SE* | χ² (df) | | *P* |
| --- | --- | --- | --- | --- | --- | --- | --- | --- |
| Intercept (control, inbred unrelated) | | | 3105.040 | | 158.510 | 383.722 (1) | | **<0.001** |
| Body length (standardised) | | | 205.430 | | 69.480 | 8.742 (1) | | **0.003** |
| Adult age at testing (standardised) | | | -261.740 | | 71.170 | 13.528 (1) | | **<0.001** |
| Temperature (warm) | | | 32.630 | | 213.630 | 0.023 (1) | | 0.879 |
| Female type (inbred related) | | | 279.200 | | 213.310 | 4.061 (2) | | 0.131 |
| Female type (outbred unrelated) | | | 406.130 | | 208.210 |  | |  |
| Temperature (warm) * Female type (inbred related) | | | -562.380 | | 291.490 | 3.795 (2) | | 0.150 |
| Temperature (warm) * Female type (outbred unrelated) | | | -194.960 | | 291.760 |  | |  |
| Random effect | Variance | *SD* | | Number of groups | | |  |  |
| Brood ID | 143114 | 378.3 | | 57 | | |  |  |
| Residual | 492561 | 701.8 | |  | | |  |  |

1. Final model excluding the non-significant interaction

|  | | | Estimate | | *SE* | χ² (df) | | *P* |
| --- | --- | --- | --- | --- | --- | --- | --- | --- |
| Intercept (control, inbred unrelated) | | | 3237.690 | | 133.360 |  | |  |
| Body length (standardised) | | | 189.960 | | 69.030 | 7.573 (1) | | **0.006** |
| Adult age at testing (standardised) | | | -262.400 | | 70.990 | 13.661 (1) | | **<0.001** |
| Temperature (warm) | | | -232.640 | | 128.770 | 3.264 (1) | | 0.071 |
| Female type (inbred related) | | | -14.000 | | 151.190 | 6.239 (2) | | **0.044** |
| Female type (outbred unrelated) | | | 318.220 | | 147.700 |  | |  |
| Random effect | Variance | *SD* | | Number of groups | | |  |  |
| Brood ID | 136300 | 369.2 | | 57 | | |  |  |
| Residual | 503812 | 709.8 | |  | | |  |  |

1. Pairwise comparison for the significant effect of female type

| Contrast | Estimate | *SE* | df | *t* ratio | *P* |
| --- | --- | --- | --- | --- | --- |
| Inbred unrelated – Inbred related | 14 | 152 | 135 | 0.092 | 0.995 |
| Inbred unrelated – Outbred unrelated | -318 | 148 | 117 | -2.146 | 0.085 |
| Inbred related – Outbred unrelated | -332 | 155 | 135 | -2.148 | 0.084 |

***Part 3 – Effect of male body size and age at testing on sperm count***


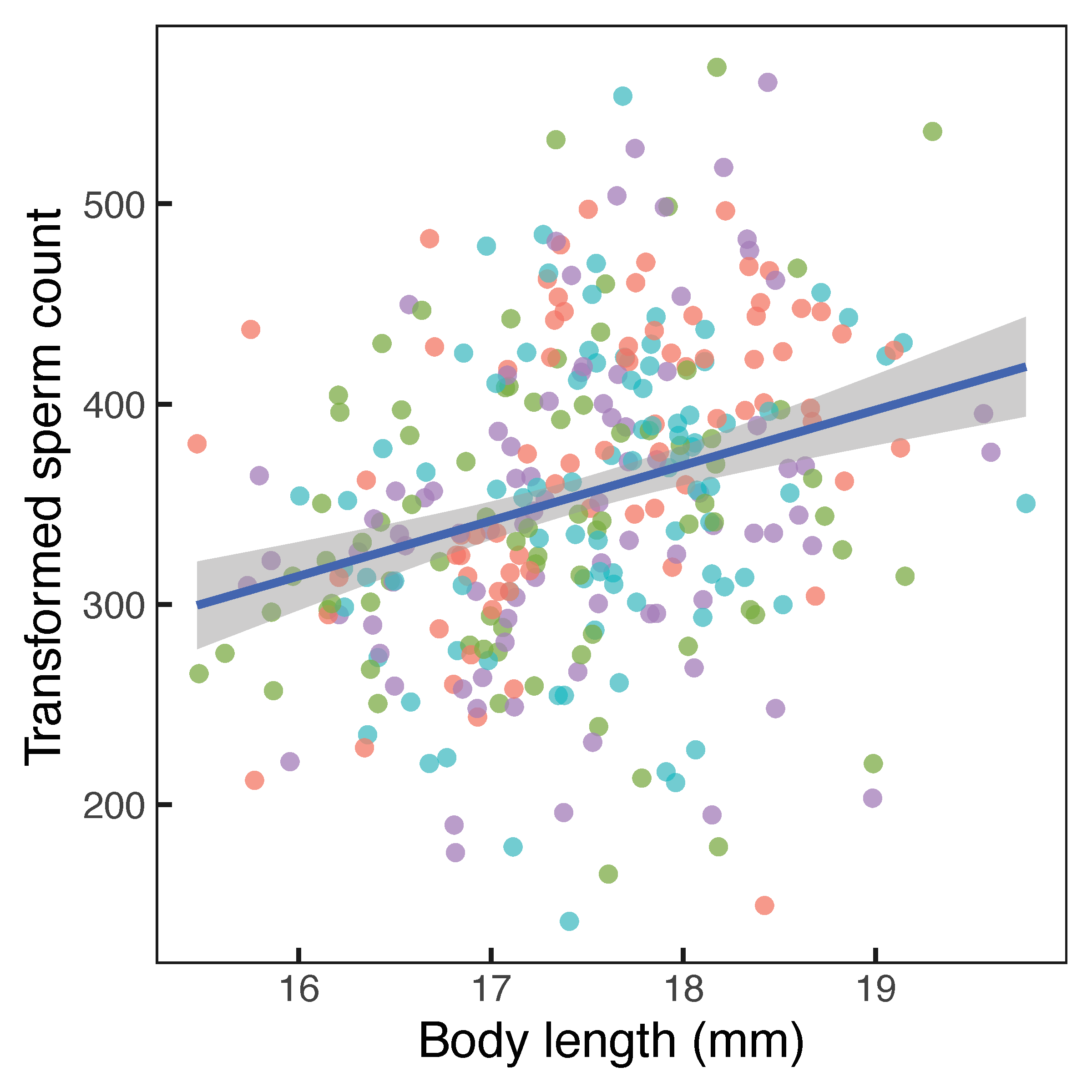


**Figure S1. Relationship between male body length and sperm count.** Colour indicates the treatment combination of male inbreeding status and rearing temperature: red = inbred-control; green = inbred-warm; blue = outbred-control; purple = outbred-warm. Body size-dependence of sperm count across all males is shown using a regression line with the 95% confidence interval.


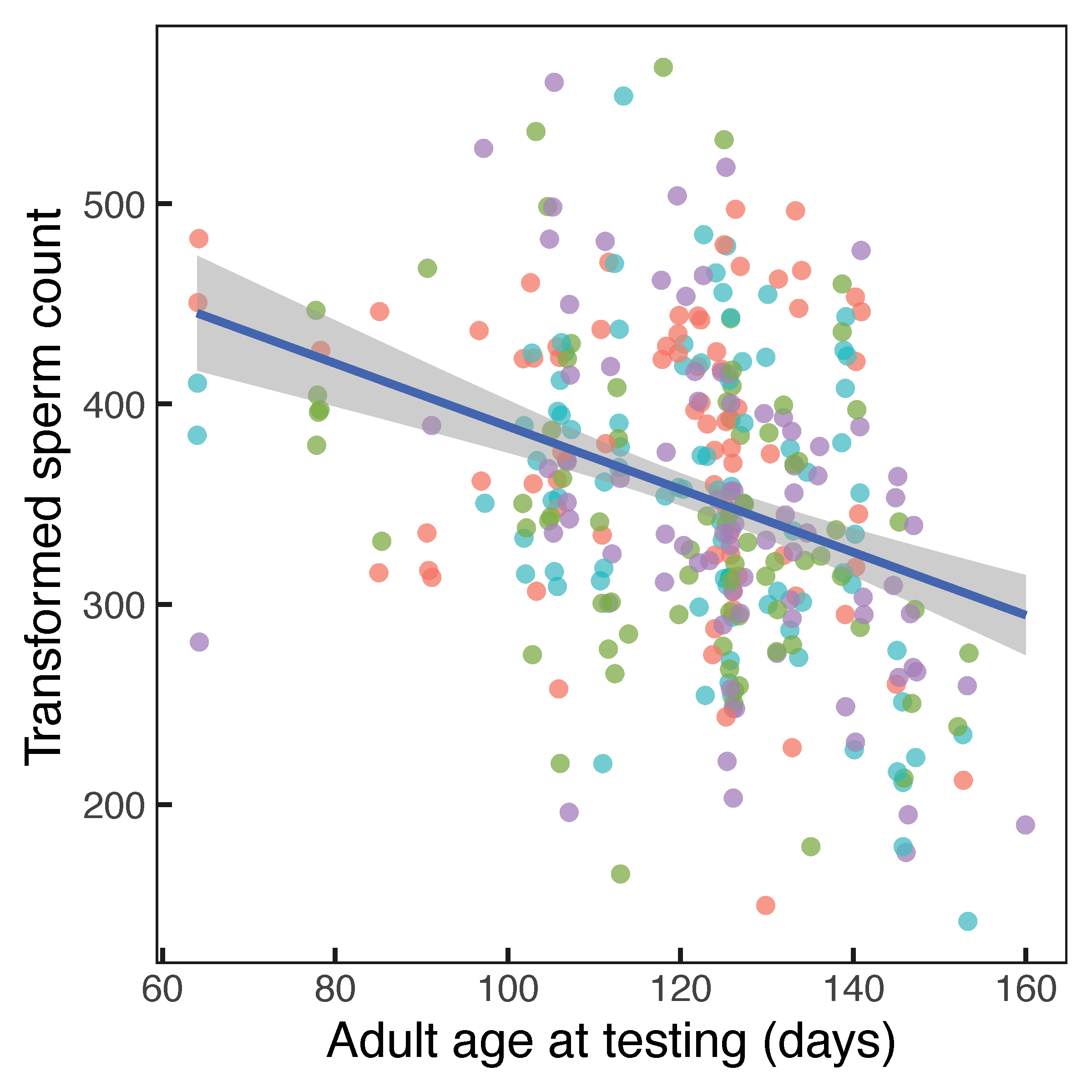


**Figure S2. Relationship between male age at testing and sperm count.** Colour indicates the inbreeding status-rearing temperature combination of male treatments: red = inbred-control; green = inbred-warm; blue = outbred-control; purple = outbred-warm. A regression line with the 95% confidence interval for all datapoints was shown.
